# Differential Vulnerability and Response to Injury among Brain Cell Types Comprising the Neurovascular Unit

**DOI:** 10.1523/JNEUROSCI.1093-22.2024

**Published:** 2024-03-28

**Authors:** Padmesh Rajput, Allison Brookshier, Shweta Kothari, Lillie Eckstein, Heather Chang, Sophie Liska, Jessica Lamb, Samuel Sances, Patrick Lyden

**Affiliations:** ^1^Department of Physiology and Neuroscience, Zilkha Neurogenetic Institute, Keck School of Medicine of USC, Los Angeles, California 90089-2821; ^2^Chinook Therapeutics, Inc., Vancouver, British Columbia V5T 4T5, Canada; ^3^Cedars-Sinai Medical Center, Los Angeles, California 90048

**Keywords:** cell death, ischemia, neurovascular unit, tolerance, vulnerability

## Abstract

The neurovascular unit (NVU) includes multiple different cell types, including neurons, astrocytes, endothelial cells, and pericytes, which respond to insults on very different time or dose scales. We defined differential vulnerability among these cell types, using response to two different insults: oxygen–glucose deprivation (OGD) and thrombin-mediated cytotoxicity. We found that neurons are most vulnerable, followed by endothelial cells and astrocytes. After temporary focal cerebral ischemia in male rats, we found significantly more injured neurons, compared with astrocytes in the ischemic area, consistent with differential vulnerability in vivo. We sought to illustrate different and shared mechanisms across all cell types during response to insult. We found that gene expression profiles in response to OGD differed among the cell types, with a paucity of gene responses shared by all types. All cell types activated genes relating to autophagy, apoptosis, and necroptosis, but the specific genes differed. Astrocytes and endothelial cells also activated pathways connected to DNA repair and antiapoptosis. Taken together, the data support the concept of differential vulnerability in the NVU and suggest that different elements of the unit will evolve from salvageable to irretrievable on different time scales while residing in the same brain region and receiving the same (ischemic) blood flow. Future work will focus on the mechanisms of these differences. These data suggest future stroke therapy development should target different elements of the NVU differently.

## Significance Statement

For decades, stroke treatments proven effective in preclinical models have failed in human clinical trials. Experimental, preclinical evaluation has focused mostly on protecting neurons, assuming that glia and vascular cells also would be protected. We now define and demonstrate differential vulnerability to insult as well as differential response to treatment among the various brain cell types comprising the neurovascular unit (NVU). Neurons, astrocytes, and endothelial cells show markedly different responses to ischemia. Ignoring differential vulnerability and treatment response may explain past clinical trial failures. Future studies should determine the mechanisms that underly differential vulnerability in the NVU.

## Introduction

The brain includes specialized cell types that function together to support different neurophysiological activities such as neurotransmission or cerebrovascular functions such as the blood–brain barrier (BBB; Wahl et al., 1988; Hawkins and Davis, 2005; Zlokovic, 2011). Brain cell types—including neurons, astrocytes, endothelial cells, pericytes, oligodendroglia, and microglia—operate together as a neurovascular unit (NVU) to respond to biochemical or molecular stimuli differently (del Zoppo, 2010; Iadecola, 2017). Brain cell types might also respond to injury (ischemia, trauma, cytotoxins) differently, yet in neurology, critical brain cell type–specific differences are generally ignored. For example, “regional selective vulnerability” to ischemia was described more than 50 years ago (Kirino and Sano, 1984; Collins et al., 1989; Mordecai et al., 1991), and our mechanistic understanding continues to enlarge (Rashad et al., 2020), yet nearly all clinical trials of cerebroprotection for stroke or hypoxic–ischemic brain injury used a single therapeutic approach, for example, a fixed treatment dose given for a fixed dosing schedule. To illustrate the risk of this fixed-dose approach, we recently showed that a treatment for cerebral hypoxia–ischemia, therapeutic hypothermia, could be optimized for targeting neuronal salvage versus BBB protection by adjusting the depth of the target temperature and the duration of treatment (Lyden et al., 2018). This optimized approach avoided coincident glial suppression during attempted cerebroprotection, resulting in an overall better outcome. We further illustrated differential drug effects by showing that some drugs protect neurons during middle cerebral artery occlusion (MCAo) at doses different from doses needed to protect BBB (Rajput et al., 2019).

Here, we define differential vulnerability as a cell type–specific biological property that results in variable resistance to injury, depending on the type of brain cell. On the other hand, regional selective vulnerability is well known and appears to depend on several factors, for example, regional cerebral blood flow (CBF) and the topographic distribution of excitotoxic synapses throughout the brain (Mordecai et al., 1991). Most importantly, in vivo the regional selective response by any one cell type depends on the response of the surrounding, supporting cell types. For example, in focal ischemia, neurons appear more vulnerable than astrocytes, which appear more vulnerable than endothelial cells, a hierarchy that likely reflects the relative distance of each cell type from the nearest microvessel (Mabuchi et al., 2005). In contrast to regional selective vulnerability, we define cell type–specific differential vulnerability in terms of each cell type's relative liability to substrate deprivation in monocellular culture (Lyden et al., 2018). In this setting, neurons are most vulnerable, but endothelial cells are next and astrocytes appear least vulnerable to substrate deprivation (Lyden et al., 2018).

Although the mechanism of cell type–specific differential response to ischemia may likely involve several linked mechanisms, we previously established a key role for thrombin cytotoxicity in mediating differential vulnerability (Rajput et al., 2014). Thrombin is a serine protease found in the serum that mediates thrombosis and neuroinflammatory responses. During ischemia, thrombin acts to kill all cells in the NVU, but neurons are most susceptible (Chen et al., 2010, 2012). We showed that thrombin cytotoxicity is mediated by the protease-activated receptor, PAR1 (Rajput et al., 2014). Drug treatments that moderate thrombin cytotoxicity proved highly cerebroprotective (Wang et al., 2013; Lyden et al., 2014, 2021; Griffin et al., 2016; Rajput et al., 2019).

Here, we seek to identify differential vulnerability using two different injury models, OGD and thrombin cytotoxicity. Then we illustrate differential vulnerability using pharmacological studies that reveal different responses to treatment interventions. We further delineate differential vulnerability using genomic analysis of each cell type's differential gene expression response to OGD. A greater understanding of brain cell type–specific responses could allow optimized treatment in which dose/timing protocols would be tailored to each therapeutic target, for example, neuronal salvage after stroke, BBB protection after global ischemic injury, or oligodendroglia salvage during demyelination. Further, these genomic studies may eventually reveal why some cell types are more resistant by identifying cellular mechanisms of injury resistance unique to the more resistant cell types.

## Materials and Methods

The Institutional Animal Care and Use Committee (IACUC) at Cedars-Sinai Medical Center (where the cell culture work was done) and Keck School of Medicine of the University of Southern California (where the animal work was done) approved all animal handling and surgery protocols. The use of laboratory animals in this study was conducted in accordance with the Guide for the Care and Use of Laboratory Animals (NRC 2011) and local Cedars-Sinai Medical Center IACUC–approved protocols.

### Reagents and antibodies

The SCH-79797 was purchased from Tocris Bioscience, catalog #1592. All other scientific grade drugs were purchased from Sigma-Aldrich. Unless otherwise specified, all cell culture reagents were purchased from Life Technologies. Timed pregnant Sprague Dawley females were purchased from Envigo Laboratories or Charles River. All antibodies were validated using known positive and negative control specimens. The primary antibodies include NeuN polyclonal antibody (Thermo Fisher Scientific: PA5-78499; concentration, 1:300) and mouse anti-human glial fibrillary acidic protein (GFAP) monoclonal antibody, unconjugated, Clone G-A-5 (Millipore: IFO3L-100UG; concentration, 1:200). The secondary antibodies include AlexaFluor 594 goat anti-rabbit IgG (H + L; Thermo Fisher Scientific: A11037; concentration, 1:600); AlexaFluor 488 goat anti-mouse IgG (H + L; Thermo Fisher Scientific: A11029; concentration, 1:600); and SYTOX Red Dead Cell Stain, for 633 or 635 nm excitation (Thermo Fisher Scientific: S34859; concentration, 0.25 µM; duration, 10 min).

### Neuronal cell culture

Primary striatal neuron cultures were prepared from E17 to E20 Sprague Dawley rats, as previously described (Rajput et al., 2014). Briefly, the striatum was carefully separated and isolated, cleaned of meninges, and finely minced. The tissue was digested in 0.25% trypsin for 5 min in a 37°C water bath with occasional gentle shaking. DNAse I was added to the cells at 37°C for another 5 min. The cells were removed, brought up to 50 ml warmed Neurobasal Plus Medium, and centrifuged for 5 min at 1,000 × *g*. The cell pellet was resuspended in neuronal complete media (Neurobasal Plus Medium from Thermo Fisher Scientific catalog #A3582901, 1% penicillin–streptomycin (Antibiotic-Antimycotic), 1% GlutaMAX, 1% B-27 Supplement, horse serum) and then filtered through a 70 µM diameter membrane, removed, resuspended, and counted for total cell concentration before plating. Neurons were plated on 96-well plates (2.5–3 × 10^4^ cells/well) coated with poly-d-lysine. After 4 d, the cells were treated with 2.5 µM 5-fluoro-2′-deoxyridine and 0.25 µM cytosine arabinoside for 24 h to suppress glial cells. Experiments were performed on confluent cultures after 10–14 d in six wells.

### Astrocyte cell culture

Primary astrocytes were isolated from P1/P2 Sprague Dawley rat brain (cortex and striatum) as described for neurons, but prior to plating, the cells were grown in T75 flasks in astrocyte complete media [DMEM (1×) + 4.5 g/L d-glucose, ʟ-glutamine, sodium pyruvate, phenol red) 1% N-2 supplement, 1% GlutaMAX, 1% penicillin–streptomycin (Antibiotic-Antimycotic), 1% sodium pyruvate, 10% FBS] at a cell density of 10^7^ cells per flask. After 3 d, the cells were shaken to remove the floating microglial cells: flasks were shaken for 2 h at 250–300 rpm, and then the media was replaced with fresh astrocyte complete media; the same process was repeated after 2–3 d. Then, cells were washed with phosphate-buffered saline (PBS), dissociated with trypsin and plated on 96-well plates at a concentration of 2.5–3 × 10^4^ cells/well. This procedure leads to a cell culture in which at least 95% of cells are astrocytes, confirmed with GFAP staining.

### Rat brain endothelial cells

Primary rat brain endothelial cells were isolated from P1 to P2 Sprague Dawley rats. Meninges were removed from the forebrain, which was then minced into small pieces in ice-cold DMEM solution and further dissociated with trituration using a 1 ml pipette. The tissue suspension was digested in 10 ml of DMEM containing collagenase (10 mg/ml) and DNAse 1 (1 mg/ml) for 1 h at 37°C. An additional 10 ml of DMEM was added to neutralize enzymes, and the suspension was centrifuged at 1,000 × *g* for 10 min. The pellet was resuspended in 25 ml of DMEM that included bovine serum albumin (BSA, 20%w/v) and centrifuged at 1,000 × *g* for 20 min at 4°C. The myelin and BSA layer were discarded, and the pellet was resuspended in 9 ml of DMEM containing 1 ml of collagenase (1 mg/ml) and 0.1 ml of DNAse I and incubated for 1 h at 37°C. Then, 10 ml of DMEM was added and the suspension was centrifuged at 250 × *g* for 10 min. The pellet was resuspended in endothelial complete media (Cell Biologics catalog #M1266PF). Cells were cultured in T25 flasks coated with collagen IV. After 2 d, the medium was changed to fresh media without puromycin. The cells from 80% confluent cultured flasks were passaged and plated on collagen IV–coated 96-well plates at a concentration of 2.5–3 × 10^4^ per well.

### Pericytes

Primary rat brain vascular pericytes were purchased from ScienCell Research Laboratories (catalog #R1200) and cultured as per the instructions provided by the manufacturer. Cells were stained with PDGFR-β and αSMA for pericytes and IBA-1 and GFAP to confirm the purity of cells.

### Oxygen–glucose deprivation

Glucose-free DMEM medium without any serum was bubbled with a gas mixture of 95% N_2_ with 5% CO_2_ for 30 min at 37°C to create an “OGD medium.” To initiate substrate deprivation in any of the four cell type cultures, the wells were washed with PBS, covered with 200 μl of OGD medium, and immediately transferred to an incubator filled with 95% N_2_/5% CO_2_ at 37⁰C. Following OGD, the cells were removed from the incubator, the OGD medium was carefully removed, and the wells were refilled with the appropriate maintenance media (complete media without serum) for the respective cell types. The plates were maintained at normal conditions for up to 24 h.

### Cell viability assay

Cytotoxicity was quantified by measuring the reduction of 3-(4,5-dimethylthiazol-2-yl)-2,5-diphenyltetrazolium bromide (MTT) to produce a dark blue formazan product (Mosmann, 1983). The colorimetric assay correlates with cellular metabolic activity due to the function and integrity of mitochondria (Berridge et al., 2005). At the end of each experiment, the cell culture medium was removed and MTT was added to each culture well at a final concentration of 0.5% MTT in PBS solution (w/v). After incubation for 3 h at 37°C, the medium was removed and gently washed with PBS. The media was then replaced with a solution containing 0.4 N HCl in 99% isopropanol for 1 h. The formation of formazan was measured by recording the absorbance at a wavelength of 570 nm and a reference at 630 nm.

### Differential response to treatment

To demonstrate the differential response to treatment among the various cell types to cytoprotectants, we cultured each cell type in well plates. For each cell type, we previously determined the amount of OGD or thrombin that would cause 50% cell death, which we called the cell type–specific LD_50_ for OGD time (in hours) or thrombin dose (units), respectively (Lyden et al., 2018). For this work, we added curves for pericytes, which showed LD_50_ values essentially identical to endothelial cells (data not shown). After reaching a 60–70% confluence, the plates were randomly assigned to one of the treatment groups. Each well plate was subject to OGD or thrombin toxicity at the cell type–specific LD_50_, during which the test agent was applied to the culture wells. The OGD LD_50_ for neurons, astrocytes, and endothelial cells/pericytes was 1.5, 6, and 3 h, respectively. The thrombin concentration LD_50_ for neurons, astrocytes, and endothelial cells/pericytes was 10, 30, and 25 units, respectively. At the end of the LD_50_ exposure, the media was changed to restore normal conditions. Cell viability and cell death were quantified 24 h after the onset of exposure. We used three forms of activated protein C (APC), which are all well-studied cytoprotectants, at 1, 10, or 100 nM. We used wild-type (WT) APC and the less-anticoagulant mutants 3K3A-APC and 5A-APC. We also used argatroban, a powerful cytoprotective direct thrombin agonist, and SCH79797, a mixed agonist/antagonist of PAR1 at 1, 10, or 50 µM. In all experiments, each drug/dose was tested in six wells and compared with OGD without any treatment; the results were averaged (SD) and normalized to non-OGD control wells, *n* = 9.

### RNAseq analysis

Primary neurons, astrocytes, endothelial cells, and pericytes were subjected to OGD or thrombin toxicity at their cell type–specific LD_50_ duration of OGD or their cell type–specific LD_50_ thrombin dose for 2 h. After exposure to one of these insults, the cells were then allowed 6 h of incubation with maintenance media to allow for gene transcription. Then, cells were lysed, and total RNA was extracted using an RNeasy mini kit (Qiagen, catalog #74104) according to the manufacturer's instructions. Library construction was performed using the Illumina TruSeq Stranded mRNA library preparation kit. Briefly, total RNA samples were assessed for concentration using the NanoDrop 8000 (Thermo Fisher Scientific) and quality using the 2100 Bioanalyzer (Agilent). One microgram of total RNA per sample was used for poly-A mRNA selection using streptavidin-coated magnetic beads. cDNA was synthesized from enriched and fragmented RNA using reverse transcriptase (SuperScript II, Invitrogen) and random primers. The cDNA was converted into double-stranded DNA, and the resulting dsDNA was enriched with PCR for library preparation. The PCR-amplified library was purified using Agencourt AMPure XP beads (Beckman Coulter). The concentration of the amplified library was measured with a NanoDrop spectrophotometer, and an aliquot of the library was resolved on an Agilent 2100 Bioanalyzer. Sample libraries were multiplexed and sequenced on a NextSeq 500 platform (Illumina) using 75 bp single-end sequencing. On average, ∼20 million reads were generated from each sample. Raw reads obtained from RNAseq were aligned to the transcriptome using STAR (version 2.5.0)/RSEM (version 1.2.25) with default parameters, using Rnor 6.0 transcriptome reference downloaded from http://uswest.ensembl.org/. Expression counts for each gene in all samples were normalized by a modified trimmed mean of the *M*-value normalization method, and unsupervised principal component analysis (PCA) was performed with DESeq2 Bioconductor package version 1.30.10 in RStudio version 1.2.5033. Each gene was fitted into a negative binomial generalized linear model, and the Wald test was applied to assess the differential expressions between two sample groups by DESeq2. Benjamini and Hochberg (B–H) procedure was applied to adjust for multiple hypothesis testing, and differential expression gene candidates were selected with a false-discovery rate (FDR) <5%. Venn plot was drawn by using interactiVenn using gene list cutoff of *p* < 0.05 for OGD LD_50_ and thrombin LD_50_ compared with control conditions. The likelihood ratio test integrated in DESeq2 was used to examine two models for the counts, a full model including all treatments (control, OGD LD_50_, thrombin LD_50_) and a reduced model without treatments to find the genes differentially expressed in any or all treatments. Differential expression genes with FDR < 1% after B–H correction were mean centered, and dendrograms were calculated by the hclust hierarchical clustering method and were plotted with heatmap3 software in the gplots package (version 3.8.1) in R version 3.6.0.

### Gene pathway analysis

To determine the genomic response of cell types to OGD, we assessed genes involved with hypoxia, oxidative stress, neuronal toxicity, and PAR-1–mediated pathways from the DEG genes. We graphed heat maps in each pathway of all four brain cell types. We used Ingenuity Pathway Analysis (http://www.ingenuity.com) to examine DEG genes in each cell type to look for the most important canonical pathways and networks associated with the gene expression changes.

### MCAo

We used the standard nylon filament model as we have published previously (Van Winkle et al., 2013). Male Sprague Dawley rats 250–300 g were purchased from Charles River and housed for a minimum of 7 d for acclimatization. Following our published protocol, the right MCA was occluded for 120 min. Anesthesia was induced with 4% isoflurane in 70:30 N_2_O:O_2_ and maintained at 1–1.5% in the same gas mix. Rats received 4 mg/kg carprofen subcutaneously before skin incision. At random, animals were occluded for 2 (*n* = 4), 4 (*n* = 4), 6 (*n* = 4), or 12 h (*n* = 5). After the prespecified occlusion time, the filament was removed to allow reperfusion for 30 min. Then the animal was deeply anesthetized with 4% isoflurane in the same gas mix and perfused via thoracotomy and cardiac puncture with 100 ml heparinized saline and 200 ml 4% paraformaldehyde. Brains were then postfixed for 24 h immersion in 4% paraformaldehyde and 48 h in 30% (w/v) sucrose. Animals that showed no neurological deficits after reperfusion were excluded from histological analysis.

### Immunohistochemistry

After fixation, brains were sectioned on a freezing microtome, and 25-µm-thick sections were stained free-floating using the antibodies listed above and SYTOX red for cell injury/death. Parallel sections were stained for TUNEL. After immunostaining, sections were mounted on gelatin double-subbed slides. Regions of interest (ROI) were selected from the transitional zone of the infarct, from a marginal zone in the ipsilateral cingulate cortex supplied by the anterior cerebral artery, and from a contralateral zone homotopic to the infarct. Three 40× high-power fields within each ROI were counted using the Cell Counter ImageJ plugin (author: Kurt De Vos). Each ROI was coded, placed in random order, and then evaluated by one investigator unaware of the animal identity, MCAo duration, or which region the ROI came from. The cell counts were then de-coded, analyzed, and graphed by a different investigator.

### Experimental design and statistical analysis

Treatment groups and cell types were all blinded and coded. The code was placed into a locked safe by one lab member not involved in cell culture, treatment, image analysis, or statistics. To assess the effect of drug treatment in each cell type, the coded cell viability results (MTT) were analyzed using two-way ANOVA and Tukey's post hoc test to correct for multiple comparisons, using cell type and drug dose as the two factors. The main effects were examined but the interaction term was tested for the presence of differential susceptibility among cell types. After all analysis was finalized, the code was removed from the safe, and drug/cell types were revealed. All other analyses are described in the results sections, as appropriate. Similarly, all brain sections were analyzed blind to occlusion duration or ROI location, and after all statistics were final, the blind was revealed. Cell counts were analyzed using two-way ANOVA for cell type and occlusion duration. Post hoc comparisons were made using Tukey's HSD. Analysis was performed with r version 4.1.2 and RStudio 2021.09.0 Build 351.

## Results

### Cell type–specific response to PAR1-targeted therapies after oxygen–glucose deprivation

**Figure 1.**
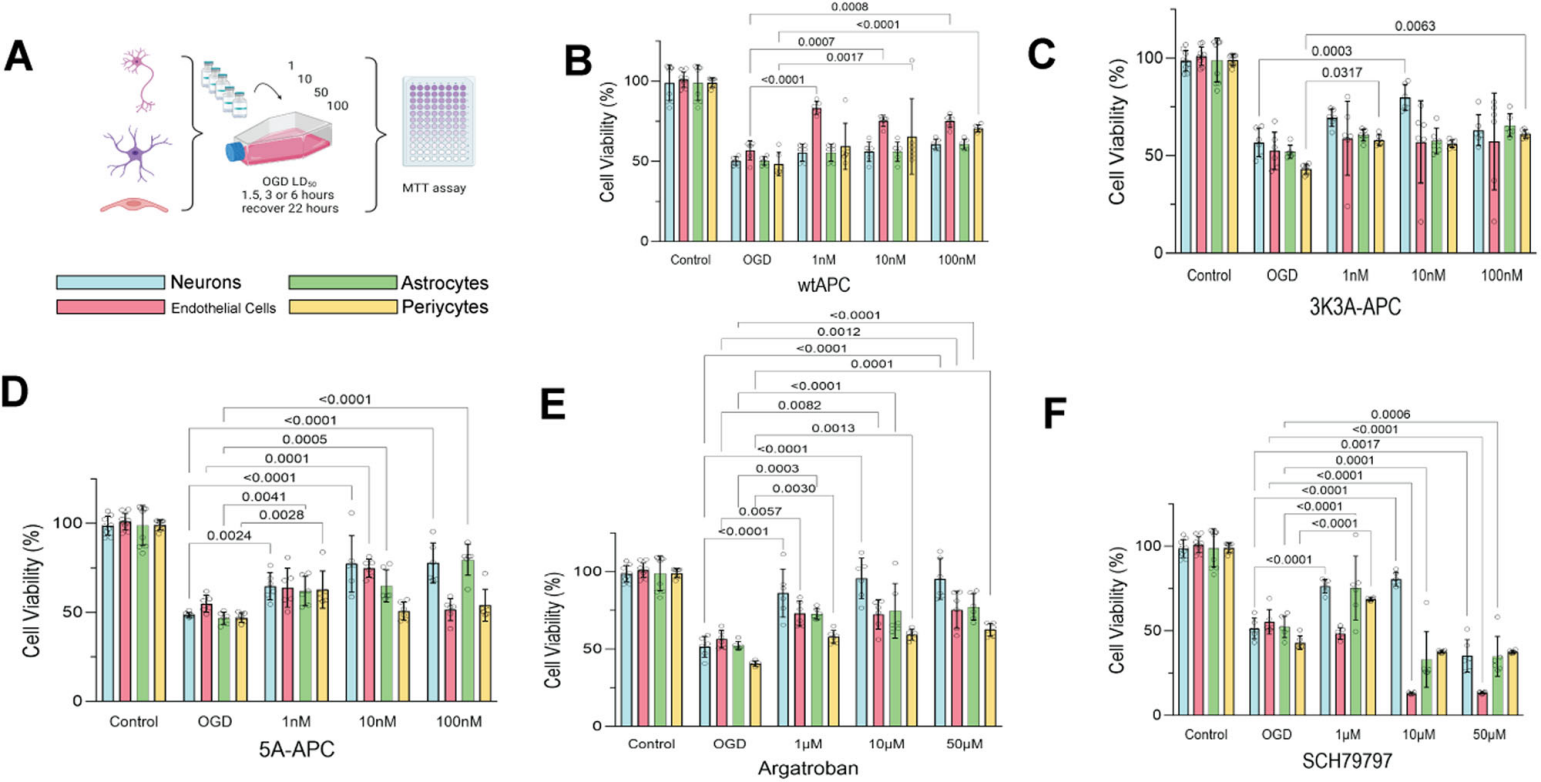
Differential response to treatment among brain cell types subjected to OGD. ***A***, Monocellular cultures were prepared and subjected to OGD duration at the LD_50_ for each cell type: neurons, 1.5 h; astrocytes, 6 h; endothelial and pericytes, 3 h. Cells were treated during the OGD with (***B***) WT-APC, (***C***) 3K3A-APC, (***D***) 5A-APC (1, 10, or 100 nM), (***E***) argatroban, or (***F***) SCH79797 (1, 10, or 50 µM). After OGD, the cells were resuspended in a cell type–specific maintenance medium and survived for 24 h, and cell viability was assessed with MTT. Each drug was tested at three different doses to establish a dose–response effect. All results were normalized to control cell cultures that were not subject to OGD. Neurons survived OGD significantly longer after 3K3A-APC and 5A-APC but not WT-APC treatment, compared with all other cell types. Endothelial cells showed significantly longer survival with the treatment of WT-APC, but not with 3K3A-APC at the doses studied. Neurons, astrocytes, and endothelial cells all survived significantly longer with various doses of argatroban. Treating cells with the PAR1 antagonist SCH79797 enhanced the survival of neurons, astrocytes, and pericytes at the 1 µM dose, but higher doses of 10 and 50 µM proved toxic to pericytes and astrocytes and especially endothelial cells. Two-way ANOVA and Tukey's test for post hoc comparisons were used to compare drug treatments within each cell type compared with OGD only.

Thrombin cytotoxicity is mediated through the PAR1 receptor (Chen et al., 2012; Rajput et al., 2014). To establish the presence of cell type–specific response to treatment and to confirm whether PAR1 plays a role in differential cell type responses to oxygen–glucose deprivation (OGD), we subjected monocellular cultures to the cell type–specific LD_50_ for OGD as determined previously and then applied cytoprotective agents known to act on thrombin or its receptor, PAR1 (Fig. 1*A*). These drugs have also been shown to be protective in animal stroke models: wild-type activated protein C (WT-APC) is cytoprotective but also an anticoagulant; the engineered mutant APCs known as 5A and 3K3A-APC retain the APC cytoprotection but without the same anticoagulant potency (Shibata et al., 2001; Fernandez et al., 2003). The PAR1 agonist/antagonist SCH79797 acts directly on the PAR1 receptor while the direct thrombin inhibitor argatroban binds to thrombin preventing it from acting on PAR1 (Ahn et al., 2000; Lyden et al., 2014). During OGD, WT-APC protected endothelial cells and pericytes at all doses tested and protected astrocytes only at the highest dose (Fig. 1*B*, main effect *F*_4,112 _= 154, *p* < 0.0001). At the doses tested here, WT-APC showed only modest protection of neurons and may require higher doses to show more neuroprotective effects. 3K3A-APC appeared to protect neurons weakly at 1 nM and powerfully at 10 nM but not at 100 nM, suggesting a dose–response effect that should be pursued (Fig. 1*C*, main effect *F*_4,112 _= 118, *p* < 0.001). 3k3a failed to protect astrocytes but protected pericytes at low and high doses. These data raise the possibility that a dose high enough to protect glia and vasculature might not protect neurons; however, this distinction requires further investigation. 5A-APC protected all cells at 1 nM but at all doses only protected neurons and astrocytes; there was no protection of pericytes and endothelial cells at higher doses (Fig. 1*D*, main effect *F*_4,112 _= 171, *p* < 0.0001). This data suggests an inverse dose–response effect such that 5A-APC doses that protect neurons might not protect vasculature and vice versa. The direct thrombin inhibitor argatroban showed a powerful cytoprotective effect on neurons subjected to OGD and was also protective of other cell types (Fig. 1*E*, main effect *F*_4,112 _= 117, *p* < 0.001). The PAR1-specific agent SCH-79797 at 1 µm protected neurons, astrocytes, and pericytes, but higher doses exacerbated injury (Fig. 1*F*, main effect *F*_4,112 _= 370, *p* < 0.0001). The data in Figure 1 demonstrate a significant difference across cell types in the NVU in their response to protection, for example, differential response to treatment. The data are consistent with a PAR1-dependent, brain cell type–specific differential vulnerability to OGD, as well as differential response to cytoprotective drugs.

### Cell type–specific response to PAR1-targeted therapies after thrombin

**Figure 2.**
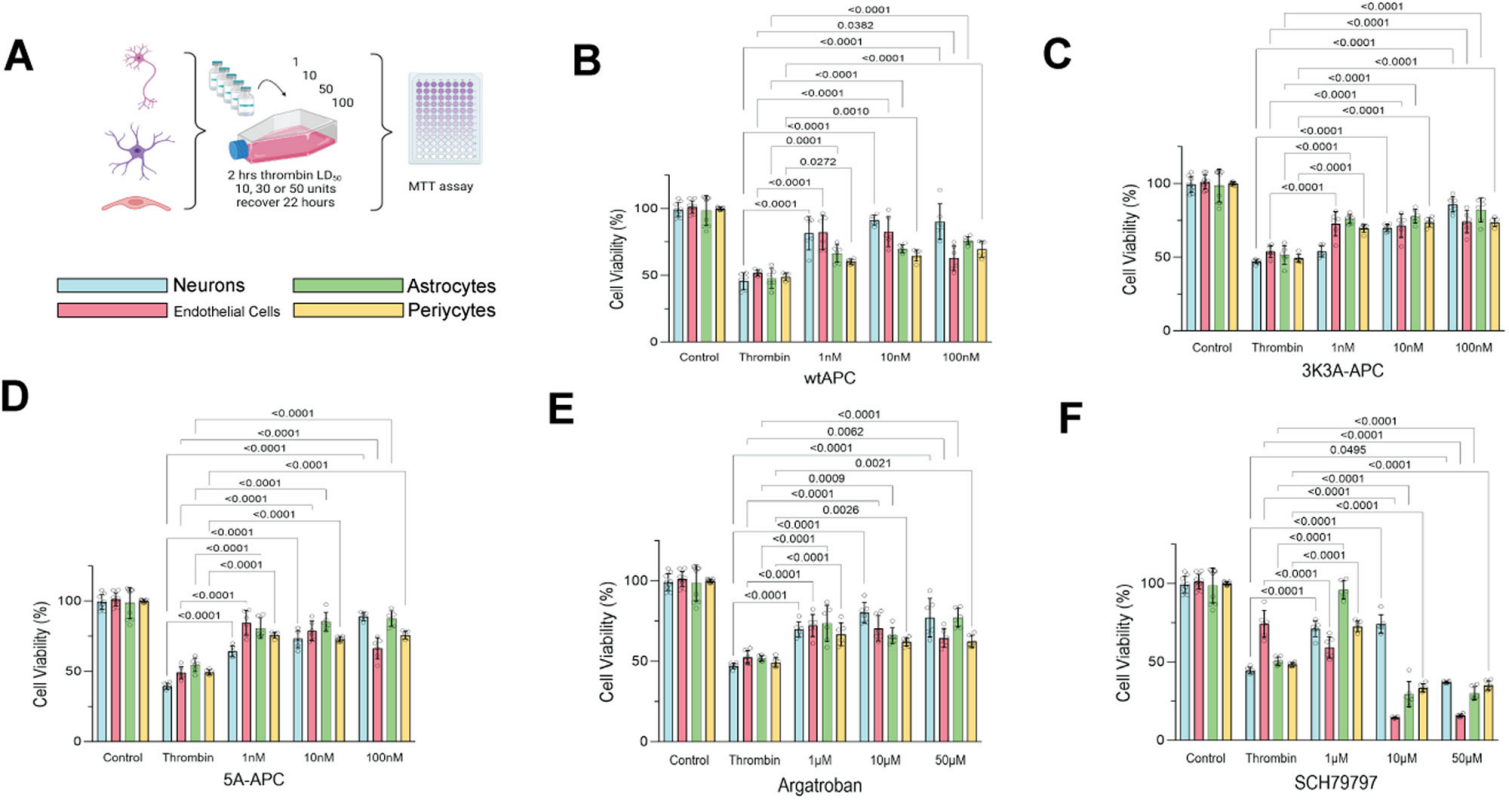
Differential response to treatment among brain cell types during thrombin cytotoxicity. ***A***, Monocellular cultures were prepared on 96-well plates and subjected to added thrombin at a concentration equal to the LD_50_ for each cell type: neurons, 10 U; astrocytes, 30 U; endothelial/pericytes, 25 U. Cells were treated during the thrombin exposure with (***B***) WT-APC, (***C***) 3K3A-APC, (***D***) 5A-APC (1, 10, or 100 nM), (***E***) argatroban, or (***F***) SCH79797 (1, 10, or 50 µM). After a 2 h dwell time, cells were resuspended in cell type–specific maintenance medium and survived until 24 h. Cell viability was measured with MTT. All four cell types showed enhanced survival from thrombin toxicity after treatment with WT-APC, the two mutant APCs, argatroban at all doses, and low-dose SCH79797. As expected, a higher dose (50 µM) of SCH79797 killed all cells. Of note, moderate dose (10 µM) SCH79797 protected neurons while killing all other cell types, another illustration of differential response. Two-way ANOVA and Tukey's test for post hoc comparisons were used for comparing different treatment effects compared with thrombin alone.

We used a second model of cell injury, thrombin cytotoxicity, to further demonstrate cell type–specific response to treatment. We tested cell type–specific responses to direct application of thrombin at the LD_50_ for each cell type as derived previously (Fig. 2*A*). We used the same drugs as used in Figure 1, which act directly on thrombin or at the PAR1 receptor. WT-APC protected all cell types at all doses (Fig. 2*B*, main effect *F*_4,112 _= 176, *p* < 0.0001). Also, 3K3A-APC and 5A-APC improved the outcome of all cell types, but there was heterogeneity in the response to different dose levels (Fig. 2*C,D*, main effect *F*_4,112 _= 300 and 300, respectively, both *p* < 0.0001). Argatroban protected all cell types from thrombin injury without showing a dose–response effect across the doses we tested, suggesting thrombin inactivation at all argatroban doses (Fig. 2*E*, main effect *F*_4,112 _= 234, *p* < 0.0001). The PAR1 agent SCH-79797 protected at a low dose of 1 µm, but higher doses of 10 and 50 µM proved toxic to all cell types (Fig. 2*F*, main effect *F*_(4,112)_ = 866, *p* < 0.0001). There were cell type–specific differences in the response to the SCH-79797: a 1 µm dose protected neurons and astrocytes; a higher dose of 10 µm protected neurons but proved cytotoxic to endothelial cells, astrocytes, and pericytes; a 50 µm dose was toxic to all cell types.

### Differential genomic response to injury using RNAseq

**Figure 3.**
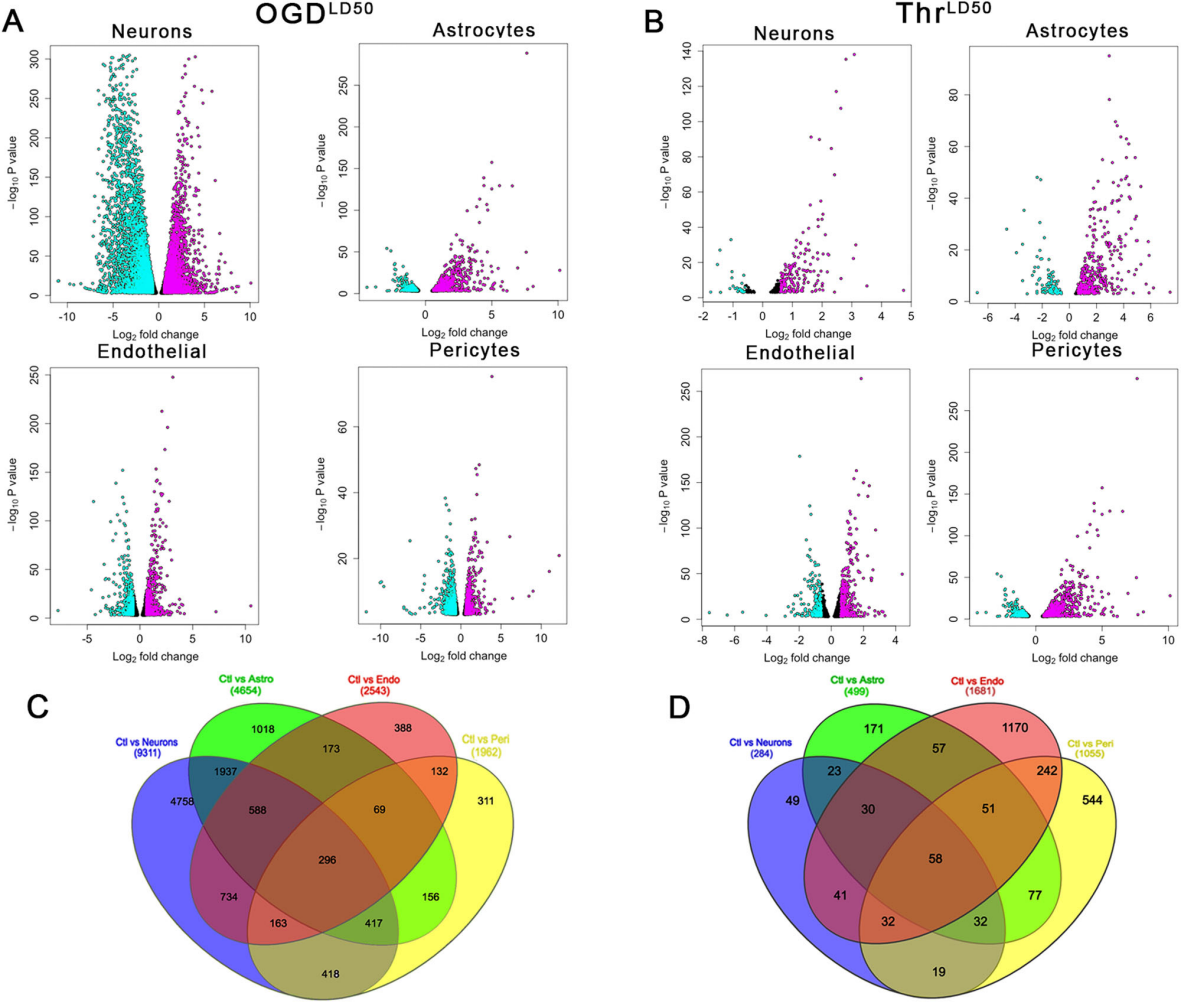
Differentially expressed genes during injury among different brain cell types. ***A***, Volcano plots illustrating changes in gene expression of different cell types subjected to cell type–specific LD_50_ OGD duration or (***B***) cytotoxic thrombin exposure. Gene expression was compared with untreated cells. Please see Extended Data Figure 3-1 for PCA. Upregulated genes (cyan dots) outnumbered downregulated genes (magenta dots). The black dots indicated genes without significant changes in expression. The *y*-axis is the log_10_ adjusted *p*-value for the difference in expression, and the *x*-axis is the log_2_ fold change. ***C***, After OGD, cell types differed in gene expression profiles, but some overlap of differentially expressed genes among all cell types could be demonstrated. Across all cell types, there were 296 shared gene expression changes. ***D***, After exposure to thrombin cytotoxicity, there were fewer changes in gene expression than after OGD, and all cell types differed from each other. There were only 58 gene expression changes shared among all cell types. The numbers in each overlap region represent the total number of downregulated plus upregulated genes.

Given the robust heterogeneity in cell type–specific response to OGD, thrombin, and cytoprotective therapies, we next sought to determine whether cell type–specific gene expression profiles after substrate deprivation were similar or different. The DEG response to cell type–specific LD_50_ OGD or cytotoxic thrombin exposure differed among the cell types (Fig. 3). Correlation and PCA indicated a high degree of gene expression similarity among biological replicates (Extended Data Fig. 3-1). Volcano plots revealed that the largest changes in DEG occurred in neurons, in which upregulated genes outnumbered downregulated genes. In other cell types, downregulated genes prevailed (Fig. 3*A,B*). Substrate deprivation with OGD caused far more DEG changes (Fig. 3*A*) than thrombin cytotoxicity (Fig. 3*B*). Using Venn diagrams (Fig. 3*C,D*), we found some overlap in DEG profiles but also considerable heterogeneity in DEG among the cell types. In response to OGD LD_50_, all four cell types shared 296 gene expression changes (Fig. 3*C*).

10.1523/JNEUROSCI.1093-22.2024.f3-1 Figure 3-1 Contrastive PCA analysis of biological replicates. Bulk RNAseq was performed on 3 replicate plates for each of 4 cell types subjected to 3 different conditions: control (Ctl), LD_50_ for OGD (hours) and LD_50_ for thrombin (Thr) cytotoxicity (units). Using contrastive PCA analysis, we verified that all 3 replicates gave similar results. Examining biplots for each cell type shows that the replicates generally group together, although not in all cases. The tight grouping reflects on the validity of the results, in that three replications yielded similar gene expression profiles. Download Figure 3-1, TIF file.

### Differential gene pathway analysis

**Figure 4.**
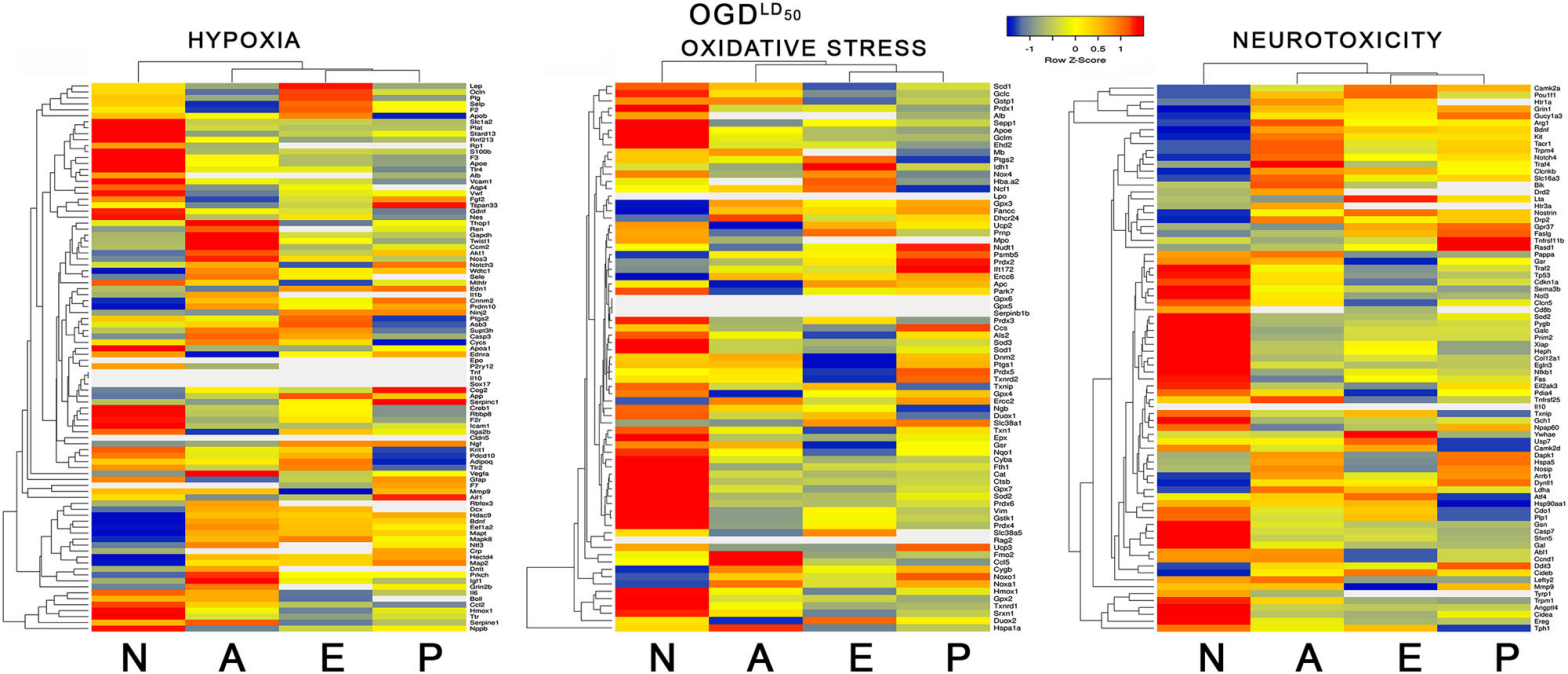
Pathway analysis shows different expression profiles among brain cell types during OGD. The gene expression profiles described in Figure 3 were mapped into three common pathway templates known to show significant changes during ischemia: hypoxia, oxidative stress, and neurotoxicity. The colors represent the *Z*-scores as shown in the legend. All four brain cell types in the NVU show considerable differences from each other in their gene expression profiles in these three pathways. Please see Extended Data Figures 4-1 and 4-2 for more information.

All four brain cell types differed considerably in their OGD responses in the pathways focussed on hypoxia response, neurotoxicity, and oxidative stress (Fig. 4). From inspection of the heat maps, neurons tended to have the most significant changes, both increased and decreased gene expression. Among all cell types, there is considerable heterogeneity in DEG profiles after OGD. Given the similar LD_50_ for endothelial cells and pericytes, the differences in their DEG profiles are striking. When filtered for gene pathways associated with stroke, the above findings were recapitulated (Extended Data Fig. 4-1).

10.1523/JNEUROSCI.1093-22.2024.f4-1 Figure 4-1 **Stroke Pathway Analysis for Differentially expressed genes among brain cell types during OGD.** The gene expression profiles described in Figure 4 were mapped into a pathway template including genes known to be relevant to ischemic stroke. Colors represent Z-scores as shown in the legend. All four cell types in the NVU show considerable differences in their gene expression profiles during OGD using this pathway. Download Figure 4-1, TIF file.

10.1523/JNEUROSCI.1093-22.2024.f4-2 Figure 4-2 **Par-1 Pathway Analysis for Differentially expressed genes among brain cell types during OGD and Thrombin cytotoxicity.** The gene expression profiles described in Figure 4 were mapped into the PAR-1 signal transduction pathway template. Colors represent Z-scores as shown in the legend. All four cell types in the NVU show considerable differences in their gene expression profiles the PAR-1 pathway during OGD and thrombin treatment. Download Figure 4-2, TIF file.

Some of the significantly upregulated stroke pathway genes in neurons included *plat* (tissue plasminogen activator), s100b, ApoE, tissue factor, VCAM1, von Willebrand's factor, GDNF, nestin, PAR1, ICAM1, proBNP, and heme oxygenase 1. Many of these proteins mediate neuronal response to injury. Krit1 strengthens cell connections. Significantly downregulated genes included, among others, histone deacetylase 9, eukaryotic translation elongation factor, BDNF, tau, NTF3, MAP kinase 8, MAP2, and a subunit of ubiquitin–protein ligase. These qualitative changes suggest an upregulation of injury response and a corresponding downregulation of structural synthesis and protein turnover, but these qualitative changes remain to be confirmed and quantified. Conversely, astrocytes upregulated stroke genes coding for neuroprotection (thimet oligopeptidase, Nos3, Akt1), BBB integrity (twist1, ccm2), or metabolism (renin, Gapdh). Astrocytes upregulated VEGF-A to promote angiogenesis and cleanup (Manoonkitiwongsa et al., 2001). Very interestingly, astrocytes significantly downregulated prothrombin, while increasing expression of the serine protease inhibitor serpine 1. Another clotting recognition molecule, the collagen receptor integrin subunit α2 was downregulated. Also downregulated were structural molecules related to BBB function (P-selectin, occludin), vasoconstriction (endothelin receptor A), and the angiogenic growth factor FGF2. Since stroke-related increases in FGF2 expression are well known, downregulation at this time point is provocative and requires confirmation. Early after astrocyte activation, they increase production of GFAP, but here, after an OGD LD_50_ of 6 h, GFAP expression was significantly downregulated.

At their OGD LD_50_, endothelial cells significantly altered expression of stroke pathway genes related to fibrinolysis (increased plasminogen, decreased serpin E-1), genes related to vasodilation (leptin, cyclooxygenase 2, amyloid precursor protein), genes related to inflammation (increased prothrombin, decreased interleukin-6 and hemoxygenase 1), and genes related to structure of the BBB (increased occludin). Pericytes seemed to emphasize cell survival and preserving or restoring BBB: Notch3, Cog2, Tspan33.

When gene expression profiles were mapped using the PAR1 signal transduction pathway (Extended Data Fig. 4-2), the cell type response to OGD or thrombin showed more similarity than the other pathways, yet considerable heterogeneity remained. Not surprisingly, neurons upregulated serpin, NFκB, PAR1, ICAM1, integrins, gap junction protein, sphingosine-1-P receptor 1, among many other proteins associated with growth factors and other aspects of cell preservation, as well as PAR1 signal transduction.

### Heterogeneity among cell death mechanisms

**Figure 5.**
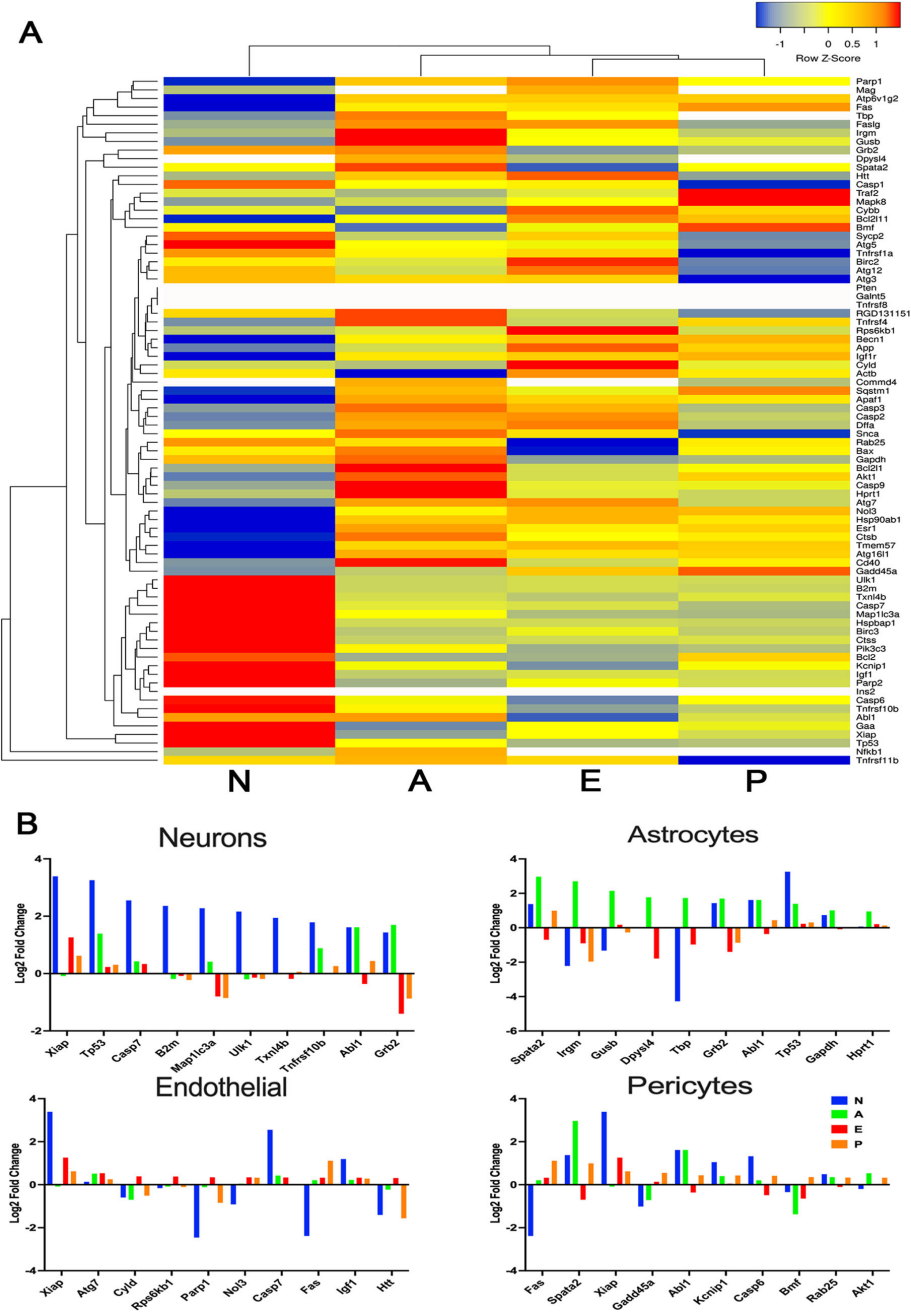
Cell death pathways activated by cell types in the NVU during OGD. ***A***, Using bulk RNAseq after pooling from cell plates subject to a 50% lethal duration of OGD, the LD_50_, gene expression results were mapped into a templated pathway including genes associated with cell death. The colors represent the *Z*-scores as shown in the legend. All four brain cell types in the NVU show considerable differences in their gene expression profiles associated with cell death. ***B***, For each cell type, the 10 genes with the largest fold change (up or down) are shown. In each panel, however, the same gene expression results from all other cell types are included for comparison. In all four subpanels, it is clearly shown that the most common gene expression changes in each cell type fail to overlap in other cell types. N, neurons; A, astrocytes; E, endothelial cells; P, pericytes.

Having demonstrated cell type heterogeneity in gene expression, we next sought to gain insight into mechanisms used by different cell types during cell death after OGD. Using a cell death pathway, the gene expression profiles differed markedly among all the NVU cell types (Fig. 5). In neurons after an LD_50_ insult (Fig. 5*B*), substantial changes were seen in genes related to apoptosis (*Xiap*, *Tp53*, *casp7*, *Abl1*, *Grb2*), neuroimmune response (*B2m*: β2 microglobulin), autophagy (*Map1lc3a*, *Ulk1*), and protection from TNF, which may indicate protection from necroptosis (*Tnfrsf10b*). These gene changes relate to several neurotoxic pathways, notably APP, TP53, and caspase 3, among others.

Other NVU cell types exhibited very different patterns of gene expression change after the OGD duration of their own LD_50_ (Fig. 5*B*). Upregulated genes in astrocytes (Fig. 5*B*, shown with corresponding changes in the other three cell types for comparison) included *Spata2*, which participates in TNFα-induced necroptosis, *Irgm* that plays a role in autophagy, *Gusb* that degrades glycosaminoglycans, *Dpysl4* that is induced by p53 to regulate energy metabolism, and four genes involved in regulating transcription in response to stress: *Tbp*, *Grb2*, *Abl1*, and *Tp53*. It is notable that neurons also increase the expression of *Tp53*. *Gapdh* and *Hprt1* are both involved in energy metabolism, as part of the astrocytes’ attempt to quickly generate energy from novel sources. These changes in astrocytes suggest roles for APP, synuclein, TP53, PARP1, and Akt.

Changes in cell death gene expression in endothelial cells and pericytes (Fig. 5) differed from each other and from the other cell types. Endothelial cells addressed autophagy with increased expression of *Atg7*, *Nol3*, and apoptosis (*Xiap*, *Nol3*, *casp7*, and *Fas*), DNA repair (*Parp1*), energy mobilization (*Igf1*), and proteolysis (*Rps6kb1*; Fig. 5*B*). In contrast, pericytes appeared to prioritize apoptosis (*Fas*, *Spata2*, *xiap*, *Casp6*, *Bmf*). Pericytes did increase the expression of genes concerned with repair (*Gadd454a*, *Abl1*, *Akt1*). The role of *Kcnip1* in pericytes is unclear.

Some genes have unknown functions (*Txnl4b*). Although some gene expression changes could be seen in common among the NVU cell types, there were striking differences. Even pericytes and endothelial cells, which exhibit a very similar ischemia tolerance, did not share many gene expression changes. On the other hand, similar gene pathways were involved in all cell types, for example, autophagy, apoptosis, and necroptosis (Fig. 5*A,B*).

### Differential vulnerability to ischemia in vivo

**Figure 6.**
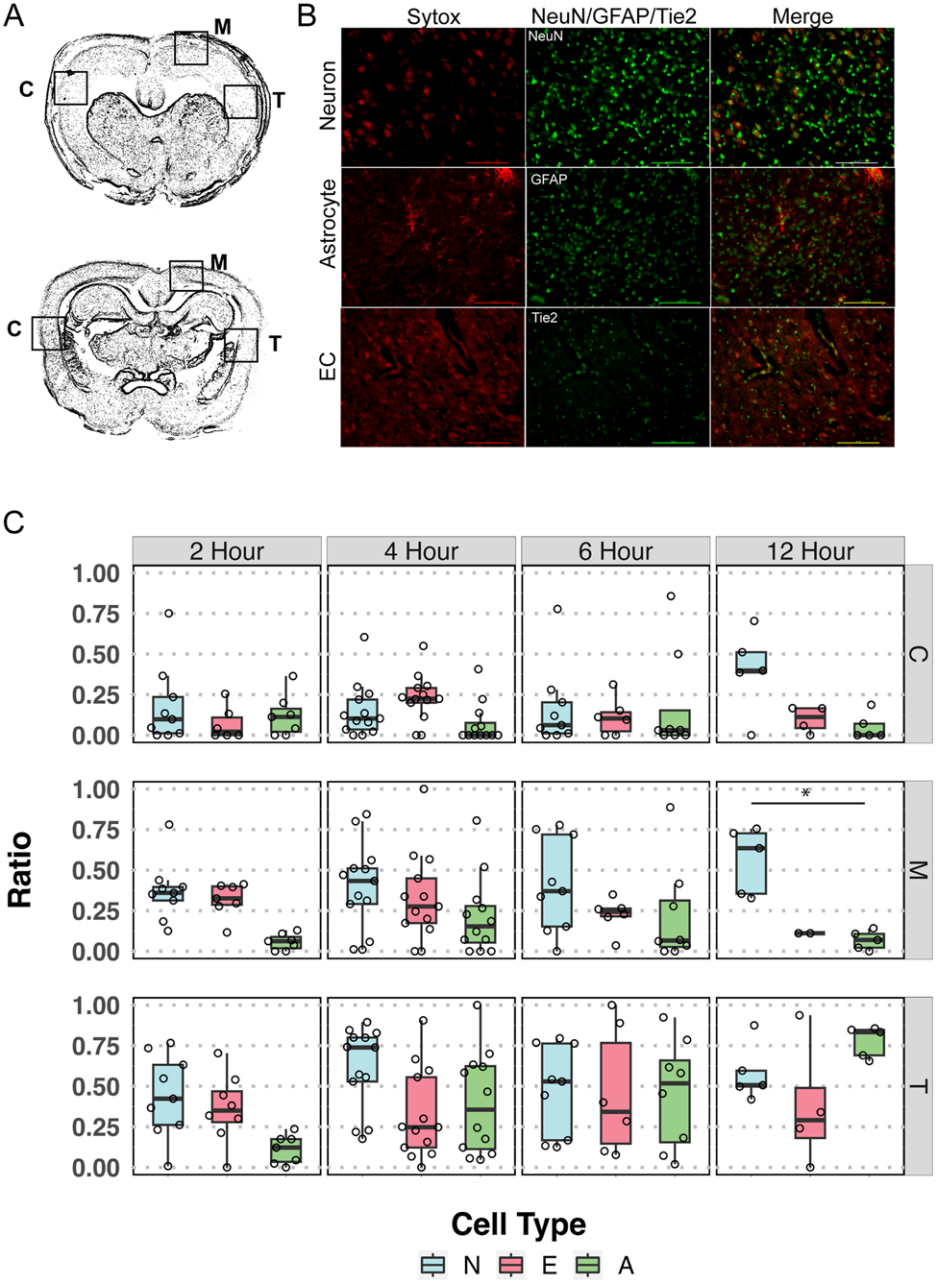
Differential vulnerability in vivo. ***A***, After temporary occlusion of the MCA for 2, 4, 6, or 12 h, rats were reperfused for 30 min and then perfused and fixed. Sections were taken from the mid-parietal cortex. ROI (*n* = 3 per zone) were strategically placed in the infarct transitional zone (T), ipsilateral marginal zone (M), or homologous contralateral zone (C), and *z*-stacks were obtained at 20× magnification. ***B***, Cells were identified using GFAP (astrocytes), NeuN (neurons), or Tie2 (endothelial cells) and stained for cell injury (SYTOX Red). Scale bar, 100 µm. ***C***, The images were coded (masked) and SYTOX-positive cells were counted by one investigator blind to occlusion duration, region, or ROI. SYTOX-positive cells were classified as positive for NeuN, GFAP, or Tie2 and summarized. After counting, the code was revealed and all counts were summarized according to occlusion duration, cell type, and region. Using two-way ANOVA to compare occlusion duration and cell type in all three counting zones, the cell type main effect was highly statistically significant in the transitional zone (*F*_2,87 _= 3.34, *p* < 0.05), marginal zone (*F*_2,84 _= 10.96, *p* < 0.001), but not in the contralateral hemisphere. Post hoc comparisons (Tukey's HSD) were limited to those of interest at *p* < 0.05. In the T zone, endothelial cells differed from neurons (*p* < 0.05) but not from astrocytes. In the M zone, endothelial cells may have differed from neurons (*p* = 0.06), and astrocytes differed from neurons (*p* < 0.0001). In the marginal zone, at 12 h neurons differed from astrocytes, *p < 0.05. N, neurons; E, endothelial cells; A, astrocytes. Please see Extended Data Figure 6-1 for a similar analysis using TUNEL staining.

Having established and characterized differential vulnerability to substrate deprivation in vitro, we sought evidence in vivo of differential vulnerability to ischemia. After 2, 4, 6, or 12 h MCAo, we counted cells labeled with the cell injury marker SYTOX Red and one of GFAP, NeuN, or Tie2 (Fig. 6). We also counted apoptotic cells using TUNEL and the same cell type–specific markers (Extended Data Fig. 6-1). In the transitional zone of the infarct (labeled “T,” Fig. 6*B,C*), all cell types showed minimal injury after 2 h ischemia, but the ratio of NeuN+/Sytox+ to total cells increased dramatically for neurons after 2 h and remained maximal at 4 and 6 h. For astrocytes, the ratio of GFAP+/Sytox+ to total cells did not reach a maximum until after 12 h ischemia. Endothelial cells were most resistant to ischemia: the ratio Tie2+/Sytox+ to total cells remained constant at all MCAo duration times, even 12 h. To assess statistical significance, we used two-way ANOVA with MCAo duration as one factor and cell type as the other. In the transition zone, the interaction term (*F*_6,87 _= 1.9, *p* = 0.09) indicated no interaction. The overall main effect of cell type (*F*_2,87 _= 3.3, *p* < 0.05) was significant, but the main effect for MCAo duration was not. There was a considerable drop off in total cell counts in the 12 h MCAo sections, however, due to ongoing destruction and phagocytosis of injured cells. In the marginal cingulate gyrus (labeled “M,” Fig. 6*B,C*), where CBF is diminished but not to infarct levels, again the neurons showed an early rise in the ratio of injured cells. The endothelial cells and astrocytes did not exhibit injury in this zone, and again, the main effect for cell type was statistically significant (*F*_2,84 _= 10.96, *p* < 0.0001). In the contralateral zone (labeled “C,” Fig. 6*B,C*) by 12 h, there were notable increases in cell injury in neurons, consistent with known transcallosal diaschisis and cell injury during unilateral injury (Andrews, 1991; Bona et al., 2019; Cheng et al., 2020). Here, the two-way ANOVA interaction term was not significant nor were the main effects. Due to the short reperfusion time of 30 min, TUNEL labeling for apoptosis did not show evidence of apoptosis after MCAo, although there were more TUNEL-positive cells of all types in the transitional zone (Extended Data Fig. 6-1).

10.1523/JNEUROSCI.1093-22.2024.f6-1 Figure 6-1 **Differential vulnerability for apoptosis in vivo .** Sections were stained with TUNEL (Terminal deoxynucleotidyl transferase dUTP nick end labeling) for apoptosis. No significant differences were noted across cell types at any occlusion duration in any regional zone. This likely reflects the very short reperfusion time of 30  min. Download Figure 6-1, TIF file.

## Discussion

We define differential vulnerability as an inherent, cell type–specific, biological property among brain cells: distinct types of brain cells respond differently to injury or treatment. Our data—pharmacologic and genomic—demonstrate a robust heterogeneity in NVU cell type–specific vulnerability to injury and cytoprotection. We demonstrated NVU differential vulnerability to OGD and found that astrocytes tolerate substrate deprivation significantly longer than endothelial cells and pericytes, both of which tolerate substrate deprivation longer than neurons (Lyden et al., 2018). This differential vulnerability was then demonstrated again by exposing cells to escalating concentrations of the cytotoxin thrombin (data not shown). The thrombin cytotoxicity curves not only replicate and confirm the presence of differential vulnerability in the NVU but also add the observation that thrombin/PAR1 partly mediates differential vulnerability.

A key role for PAR1 mediating differential vulnerability was demonstrated in response to various PAR1 acting cytoprotectants known to ameliorate the impact of OGD (Fig. 1) and agents targeting thrombin cytotoxicity (Fig. 2). To further support the existence and definition of differential vulnerability, we then found considerable heterogeneity among NVU cell types in gene expression responses to OGD and thrombin administration (Figs. 3, 4). At a duration of OGD that kills half of the cells—the cell type–specific OGD LD_50_—all four NVU cell types become committed to 24 h cell death, but the gene expression profile differed for each type (Fig. 5). Taken together, our data plausibly confirm the presence differential vulnerability as an inherent biological property of the elements of the NVU that can be observed using in vitro cell culture. Further, PAR1 signaling plays a role in mediating a differential response to injury across NVU cell types.

Differential vulnerability is not the same phenomenon as the previously described regional selective vulnerability. The in vivo “selective vulnerability” of neurons in response to hypoxic–ischemic injury was described in the CA1 and CA3 layers of the hippocampus, layers 4–6 of the cerebral cortex, and the Purkinje cell layer of the cerebellum (Haymaker and McMenemey, 1961; Kirino and Sano, 1984; Collins et al., 1989). Regional selective vulnerability appears related to the greater density of glutamatergic versus GABAeric receptors in vulnerable regions (Collins et al., 1989; Sternau et al., 1989; Mordecai et al., 1991) or to watershed effects in the microcirculation (Mabuchi et al., 2005), among other possible explanations (Rashad et al., 2020). Regional selective vulnerability also reflects the susceptibility to ischemia of NVU cells while surrounded by all other elements of the NVU. Differential vulnerability as we define it refers to inherent, cell type–specific death pathways activated at different intervals in different cell types (Fig. 5). In understanding how the brain responds to global or focal ischemia, *both* selective regional and differential vulnerabilities are important.

We previously showed significant differences between neuronal injury and endothelial damage during MCAo (Rajput et al., 2019). Here, our data suggest that astrocytes withstand ischemic insult in the penumbra (labeled “T,” Fig. 6*C*) much longer than neurons. This finding suggests the existence of differential vulnerability in vivo. Endothelial cells exhibited the greatest resistance to ischemia, likely due to their proximity to residual blood flow (Mabuchi et al., 2005).

Converging evidence from multiple laboratories supports the differential vulnerability and response to injury concept. Cell type–specific mechanisms in response to global circulatory arrest have been reviewed, but without the time course detailed by us here (Daniele et al., 2021). Using cell-selective biotinylation, the mouse proteomes of neurons and astrocytes were shown to differ in a region-specific manner (Rayaprolu et al., 2022). The transcriptome differs among eight cell types in the mouse NVU (Zhang et al., 2014). In human cerebral vasculature, different vascular cell types (endothelial cells, mural cells, and perivascular fibroblasts) can be distinguished from astrocytes based on differential gene expressions and response to neurodegenerative injury (Huntington's disease; Garcia et al., 2022). A cell type–specific hierarchy of vulnerability to toxin exposure has been documented (Luo et al., 2020). Selective neuronal vulnerability has also been described, in which different neuronal populations in the brain exhibit differential vulnerability to ischemia, and several mechanisms proposed (Centonze et al., 2001; Wang and Michaelis, 2010; Rashad et al., 2020).

The data presented here have considerable basic and translational implications. Previous recognition of the penumbra and the time-dependent evolution of penumbra to core (cell death) in the NVU improved our understanding of stroke and ischemic brain injury considerably (Hossmann, 1994; Sharp et al., 2000; Bai and Lyden, 2015; Iadecola, 2017; Lyden et al., 2018). Our present data refine and extend that understanding by adding the differential vulnerability among cell types that constitute the NVU. The concept of differential vulnerability predicts that different elements of the NVU will evolve from salvageable to irretrievable on different time scales, while residing in the same brain region and receiving the same (ischemic) blood flow (Fig. 6). In the penumbra, for example, the different populations of cell types are predicted to be evolving toward core infarction at different rates, as supported by our in vivo data (Fig. 6). This prediction remains to be confirmed, however, in additional models.

When approaching stroke treatment, the marked differential response of brain cell types to pharmacotherapy must guide and influence further preclinical development (Lyden et al., 2021). We previously demonstrated that cytoprotective treatment could target neurons and endothelial cells differently, thus demonstrating proof of concept (Rajput et al., 2019). Furthermore, drugs might protect one cell type but harm another cell type, at the same dose. We showed that although 1 µM of the PAR1 agonist SCH79797 protected neurons, astrocytes, and pericytes, at higher doses the same agent proved cytotoxic (Fig. 1). It is particularly illuminating that 10 µm SHC79797 protected neurons and simultaneously injured endothelial cells and astrocytes. These data suggest that when designing cytoprotective therapies directed at the brain during ischemia, investigators need to pay particular attention to dose selection and timing, to optimize protective effects without inadvertently causing harm to different cell types.

Using a different class of cytoprotectants, PAR1 acting agents, we further demonstrated a cell-specific response to injury and cytoprotection in the brain. Previously we demonstrated that WT-APC, 3K3A-APC, and 5A-APC act differently on the BBB and neurons during focal cerebral ischemia in rats (Rajput et al., 2019). These cell type–specific responses are recapitulated in the present data (Figs. 1, 2). WT-APC protected neurons and endothelial cells very well but less so the other cell types during OGD (Fig. 1*B*). The engineered APC molecules, 3K3A-APC protected neurons but with little protection of other cell types, while 5A-APC protected neurons and astrocytes (Fig. 1*C,D*). The 3K3A and the 5A mutations were designed to reduce the anticoagulant properties of APC, without any impact on the cytoprotective effects (Mosnier et al., 2007).

Beyond therapeutic targeting, differential vulnerability implies a fundamental biological concept: not only do cell types in the NVU respond to OGD on differing time scales, but it also appears that each cell type in the NVU accesses different cell death gene expression profiles (Fig. 5*A*). In other words, the differential resistance to OGD or thrombin cytotoxicity may arise out of the unique repertoire of molecular and biochemical responses found in each cell type (Zhang et al., 2014; Rayaprolu et al., 2022). For example, when subject to an OGD LD_50_ of 1.5 h, neurons upregulate genes associated with apoptosis, autophagy, and transducing TNF signaling (Fig. 5*B*). In contrast, after an OGD LD_50_ of 6 h, astrocytes increased expression of genes related to cell repair and salvage in addition to necroptosis and autophagy. Many of the gene responses we found reflect pathways that are not yet fully defined; hence, the roles played by individual genes cannot be inferred fully from our data, but comparing the gene expression profiles among cell types in the NVU clearly demonstrates that very different pathways to cell death exist in each of the NVU cell types.

Important limitations of our data should be noted. We raised monocellular cultures to determine the responses of pure cell types; in the functioning brain, all cell types interact with each other, certainly altering cell-specific responses to injury. Another limitation is that we gave fixed doses of each drug to demonstrate the cell type–specific responses—since we did not titrate each drug to its most effective dose in each cell type, we cannot infer relative potencies or efficacy across the various drugs. We used what we believe are PAR1 acting reagents, but there may be other receptors that are potential contributors to signaling via cross-talk or amplification of the primary APC effect on PAR1 and PAR3 (Cao et al., 2010; Minhas et al., 2010) Although this study was conducted in accordance with most commonly accepted guidelines for good laboratory practice—including blinding, randomization, and scrupulous accounting of all subjects—our conclusions do require further confirmation.

In conclusion, we defined and demonstrated differential vulnerability in the NVU. We demonstrated that PAR1 plays a role in mediating differential vulnerability to OGD and thrombin-induced injury. Different cell types in the NVU display differential responses to pharmacotherapy. Underlying these differential responses, we documented considerable gene expression heterogeneity among brain cell types responding to injury. These findings should be confirmed and could eventually profoundly influence the design of cerebroprotective clinical trials.
