# Supplementary figures and images for: Differential Vulnerability and Response to Injury among Brain Cell Types Comprising the Neurovascular Unit
Source: J Neurosci. 2024 Mar 28;44(22):e1093222024. doi: 10.1523/JNEUROSCI.1093-22.2024 (PMC11140689; doi:10.1523/JNEUROSCI.1093-22.2024)

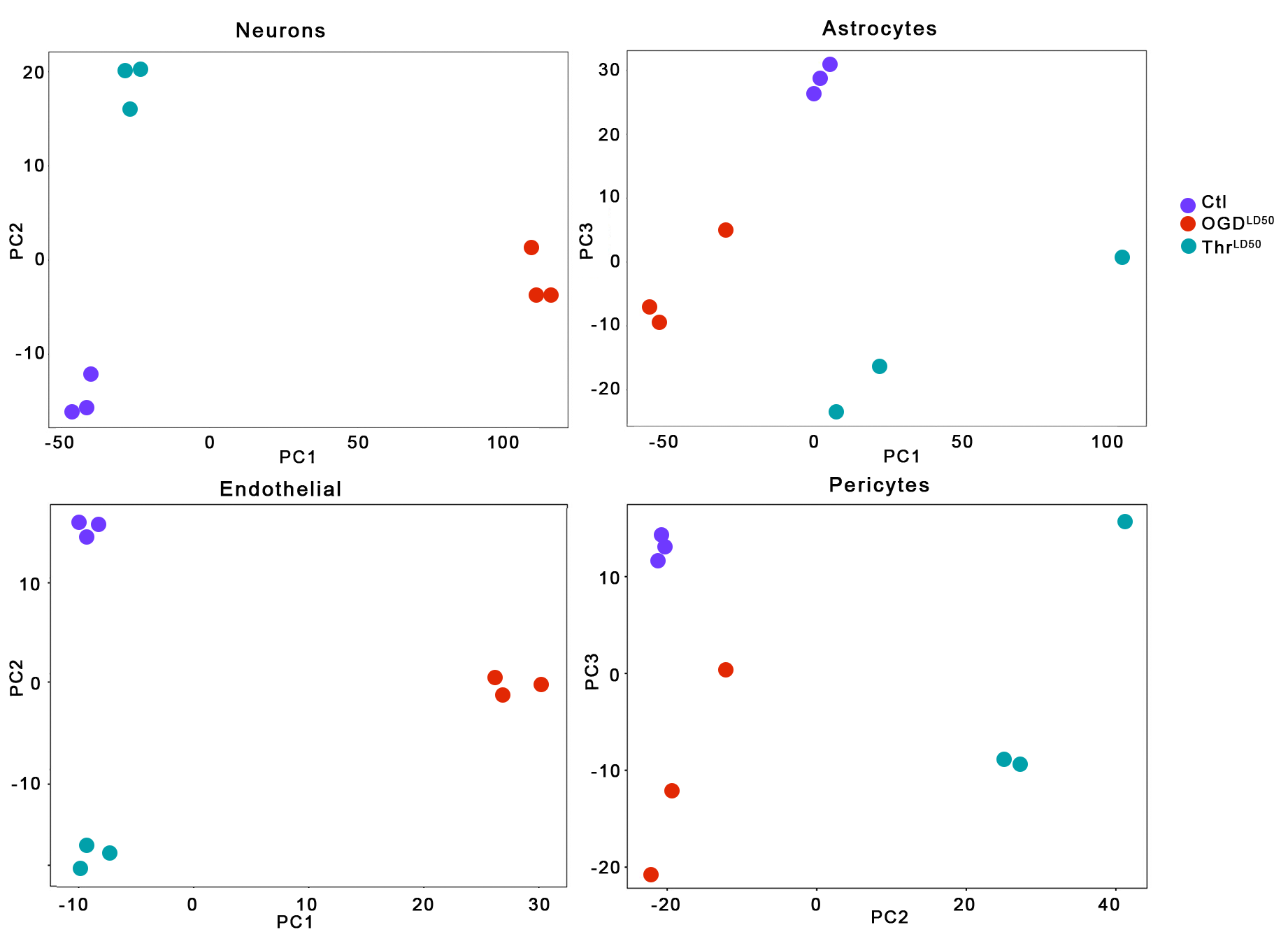

Supplement: Figure 3-1 — Contrastive PCA analysis of biological replicates. Bulk RNAseq was performed on 3 replicate plates for each of 4 cell types subjected to 3 different conditions: control (Ctl), LD50 for OGD (hours) and LD50 for thrombin (Thr) cytotoxicity (units). Using contrastive PCA analysis, we verified that all 3 replicates gave similar results. Examining biplots for each cell type shows that the replicates generally group together, although not in all cases. The tight grouping reflects on the validity of the results, in that three replications yielded similar gene expression profiles. Download Figure 3-1, TIF file. [file jneuro-44-e1093222024-s001.tif]

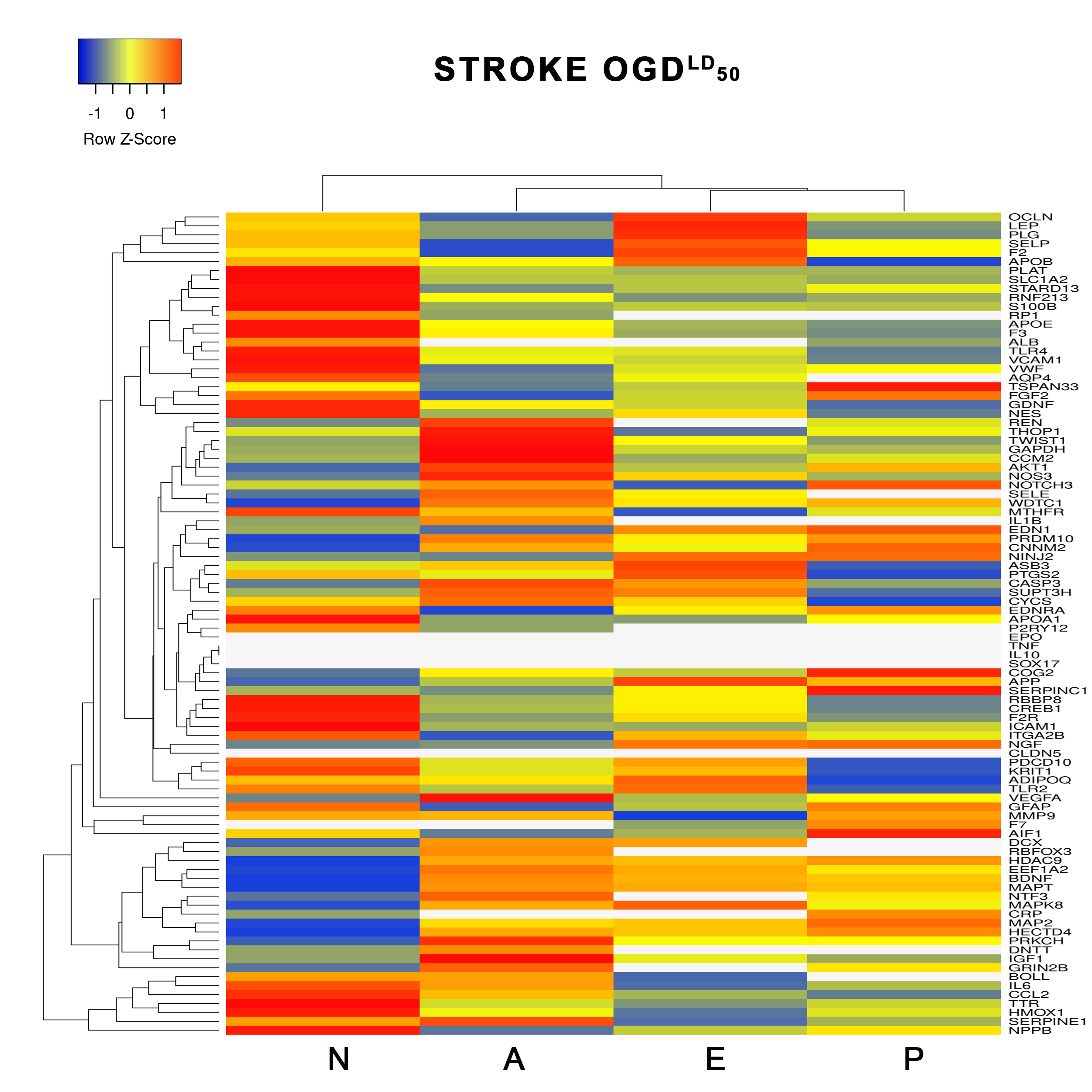

Supplement: Figure 4-1 — Stroke Pathway Analysis for Differentially expressed genes among brain cell types during OGD. The gene expression profiles described in Figure 4 were mapped into a pathway template including genes known to be relevant to ischemic stroke. Colors represent Z-scores as shown in the legend. All four cell types in the NVU show considerable differences in their gene expression profiles during OGD using this pathway. Download Figure 4-1, TIF file. [file jneuro-44-e1093222024-s002.tif]

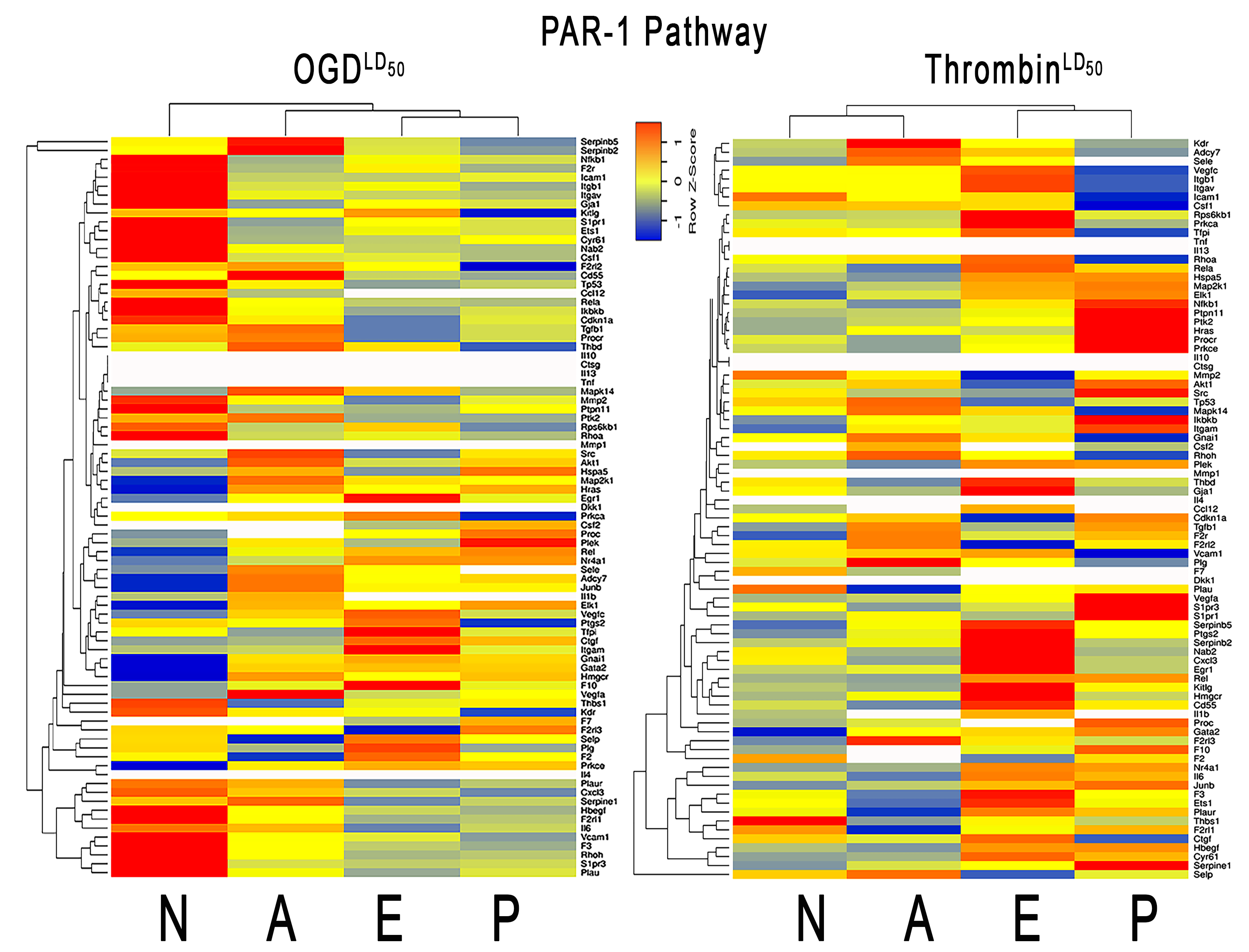

Supplement: Figure 4-2 — Par-1 Pathway Analysis for Differentially expressed genes among brain cell types during OGD and Thrombin cytotoxicity. The gene expression profiles described in Figure 4 were mapped into the PAR-1 signal transduction pathway template. Colors represent Z-scores as shown in the legend. All four cell types in the NVU show considerable differences in their gene expression profiles the PAR-1 pathway during OGD and thrombin treatment. Download Figure 4-2, TIF file. [file jneuro-44-e1093222024-s003.tif]

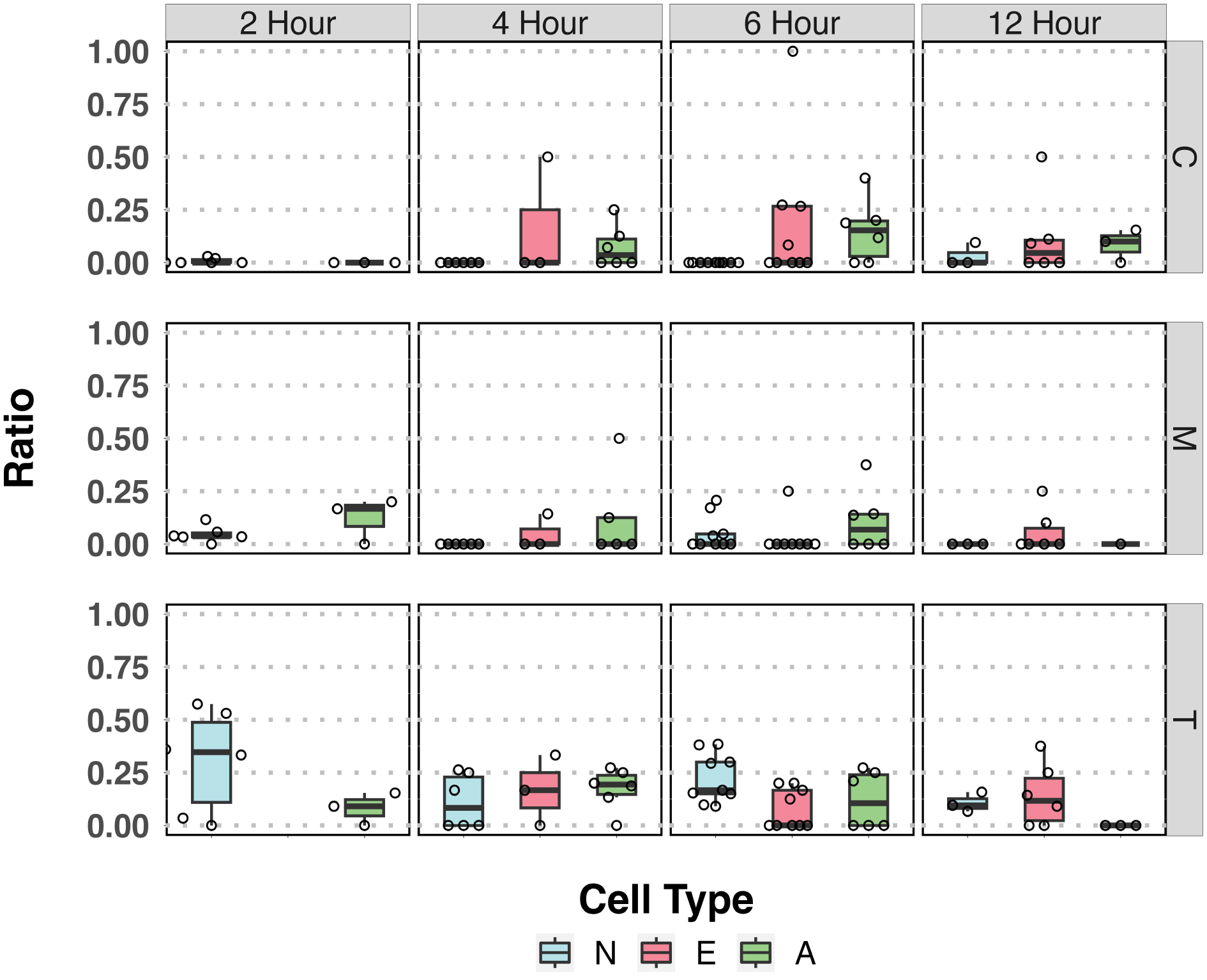

Supplement: Figure 6-1 — Differential vulnerability for apoptosis in vivo . Sections were stained with TUNEL (Terminal deoxynucleotidyl transferase dUTP nick end labeling) for apoptosis. No significant differences were noted across cell types at any occlusion duration in any regional zone. This likely reflects the very short reperfusion time of 30 min. Download Figure 6-1, TIF file. [file jneuro-44-e1093222024-s004.tif]
